# Introduction of mobile phones for use by volunteer community health workers in support of integrated community case management in Bushenyi District, Uganda: development and implementation process

**DOI:** 10.1186/1472-6963-14-S1-S2

**Published:** 2014-05-12

**Authors:** David Katuruba Tumusiime, Gad Agaba, Teddy Kyomuhangi, Jan Finch, Jerome Kabakyenga, Stuart MacLeod

**Affiliations:** 1 Mbarara University of Science and Technology, Mbarara, Uganda; 2 Child & Family Research Institute, Vancouver, British Columbia, Canada; 3 Department of Pediatrics, University of British Columbia, Vancouver, British Columbia, Canada

**Keywords:** mobile phone, child health, pediatric therapeutics, integrated community case management, community health worker, Uganda, téléphone mobile, santé des enfants, thérapeutique pédiatrique, gestion communautaire intégrée des cas, travailleur en santé communautaire, Ouganda

## Abstract

**Background:**

A substantial literature suggests that mobile phones have great potential to improve management and survival of acutely ill children in rural Africa. The national strategy of the Ugandan Ministry of Health calls for employment of volunteer community health workers (CHWs) in implementation of Integrated Community Case Management (iCCM) of common illnesses (diarrhea, acute respiratory infection, pneumonia, fever/malaria) affecting children under five years of age. A mobile phone enabled system was developed within iCCM aiming to improve access by CHWs to medical advice and to strengthen reporting of data on danger signs and symptoms for acutely ill children under five years of age. Herein critical steps in development, implementation, and integration of mobile phone technology within iCCM are described.

**Methods:**

Mechanisms to improve diagnosis, treatment and referral of sick children under five were defined. Treatment algorithms were developed by the project technical team and mounted and piloted on the mobile phones, using an iterative process involving technical support personnel, health care providers, and academic support. Using a purposefully developed mobile phone training manual, CHWs were trained over an intensive five-day course to make timely diagnoses, recognize clinical danger signs, communicate about referrals and initiate treatment with appropriate essential drugs. Performance by CHWs and the accuracy and completeness of their submitted data was closely monitored post training test period and during the subsequent nine month community trial. In the full trial, the number of referrals and correctly treated children, based on the agreed treatment algorithms, was recorded. Births, deaths, and medication stocks were also tracked.

**Results and Discussion:**

Seven distinct phases were required to develop a robust mobile phone enabled system in support of the iCCM program. Over a nine month period, 96 CHWs were trained to use mobile phones and their competence to initiate a community trial was established through performance monitoring.

**Conclusion:**

Local information/communication consultants, working in concert with a university based department of pediatrics, can design and implement a robust mobile phone based system that may be anticipated to contribute to efficient delivery of iCCM by trained volunteer CHWs in rural settings in Uganda.

## Abstract (fr)

**Résumé:**

**Contexte**

Une abondante documentation suggère que les téléphones mobiles sont susceptibles d’améliorer la survie des enfants et la gestion des cas d’enfants gravement malades en Afrique rurale. La stratégie nationale du ministère de la Santé de l’Ouganda appelle le recrutement de travailleurs en santé communautaire bénévoles aux fins de la mise en oeuvre de la gestion des cas intégrée en milieu communautaire pour les affections largement répandues (diarrhée, infection aiguë des voies respiratoires, pneumonie, fièvre, paludisme) chez les enfants de moins de cinq ans. Un système reposant sur l’utilisation de téléphones mobiles a été mis sur pied dans le cadre du programme de gestion des cas intégrée afin d’améliorer l’accès des travailleurs en santé communautaire à des conseils médicaux ainsi que la divulgation de renseignements au sujet des signes et symptômes à surveiller chez les enfants de moins de cinq ans gravement malades. Les étapes importantes de l’élaboration et de la mise en oeuvre de l’intégration des téléphones mobiles à la gestion des cas intégrée en milieu communautaire sont décrites ci-dessous.

**Méthodes**

Des mécanismes visant à améliorer l’établissement d’un diagnostic, le traitement et l’aiguillage pour les cas d’enfants malades de moins de cinq ans ont été définis. Des algorithmes de traitement ont été mis au point par l’équipe technique du projet et installés et mis à l’essai sur les téléphones mobiles dans le cadre d’un processus itératif englobant du personnel de soutien technique, des fournisseurs de soins de santé et du soutien de chercheurs. Équipés d’un manuel de formation expressément conçu pour le projet, les travailleurs ont suivi une formation intensive de cinq jours au cours de laquelle ils ont appris à établir des diagnostics en temps opportun, à reconnaître les signes cliniques de problèmes, à communiquer les mesures d’aiguillage requises et à entreprendre le traitement au moyen des médicaments essentiels adéquats. Le rendement des travailleurs ainsi que l’exactitude et l’exhaustivité des données qu’ils soumettaient ont été étroitement suivis pendant une période pilote suivant la formation, puis pendant un essai d’une durée de neuf mois. Au cours de cet essai, on a enregistré le nombre d’enfants qui ont été aiguillés et traités adéquatement en fonction des algorithmes de traitement retenus. On a aussi noté le nombre de naissances, le nombre de décès et les stocks de médicaments.

**Résultats et discussion**

Sept phases distinctes ont été nécessaires pour mettre sur pied un système robuste reposant sur l’utilisation de téléphones mobiles qui puisse soutenir la gestion des cas intégrée en milieu communautaire. Ces phases ont pris fin après une période de neuf mois. Au total, 96 travailleurs en santé communautaire ont reçu une formation sur l’utilisation de téléphones mobiles, et l’on a déterminé leur aptitude à entreprendre un essai en milieu communautaire en suivant leur rendement.

De concert avec un département universitaire de pédiatrie, des consultants en information et en communications de la région peuvent concevoir et mettre en oeuvre un système robuste fondé sur l’utilisation de téléphones mobiles pouvant contribuer à la prestation efficace, par des travailleurs en santé communautaire bénévoles formés dans les zones rurales de l’Ouganda, de la gestion des cas intégrée en milieu communautaire.

## Background

A growing body of literature attests to the potential for improved integrated child health care through application of mobile health technology. Such advances promise to be particularly advantageous in rural and isolated districts in low income countries [1-7].

Uganda is a highly rural society with more than 80% of its population residing in non-urban areas. It has one of the highest fertility rates in the world (6.1 in 2010) and in 2010 there were 6.47 million children under five out of a total population of 33.43 million [8]. Under these circumstances, there are large numbers of young children suffering from acute illnesses residing in rural and isolated areas.

The latest figures (2011) indicate an under five mortality rate for Uganda of 90 [9]. Future improvement in child mortality statistics will depend on provision of sound information to guide treatment and referral practice, to community health workers (CHWs; village health team volunteers) who are the first point of contact with sick children in Uganda. Ugandan CHWs are currently being trained to support Integrated Community Case Management (iCCM) [10] and to foster the World Health Organization policy of community-level Integrated Management of Childhood Illness (IMCI) [11]. In Uganda and in other African countries, the CHW system has demonstrated its utility as a low cost measure for delivery of basic health supports to citizens living in rural and isolated areas [12]. The success of this program in Bushenyi District, where the present study was conducted, has been described previously by the Healthy Child Uganda program which has now been operating for more than ten years [13-15].

Over the past 10 years Uganda has seen a revolution in the use of mobile phones such that most villages are now connected, at least potentially, to the services available in health centres (levels I-IV) and in district/regional referral hospitals. The program described in this and related papers [16,17] originated with the hypothesis that the communication system linking health volunteers to higher level health care could be substantially improved. It was postulated that the enhanced flow of information and counseling to CHWs would improve diagnosis and the timely referral of children under five years of age with life threatening illnesses (diarrhea, acute respiratory infection [ARI], pneumonia, fever/malaria) to appropriate levels of care with corresponding improved outcomes, reductions in morbidity and mortality.

The design/implementation process described in this paper also aimed at supporting the use of basic mobile phones in the hands of CHWs in the field to enable a more comprehensive programmatic initiative focused on improved drug therapy [16,17]. The overall project design incorporated an expectation that the timely availability of information on shortages of medicines essential for treatment of childhood illnesses would prove effective in improving supply chain management and preventing stockouts, which are often the cause of failure in management of childhood infections at a village level, and also allow for the timely referral of sick children to more advanced health care facilities.

The choice of Bushenyi District for this demonstration model was based on the ongoing experience of area residents, caregivers, and academic clinicians with Healthy Child Uganda [13-15]. The topography and demography of the region are ideally suited for evaluation of the impact of a mobile phone-enabled extension of child health services.

A detailed report on efficacy of the iCCM program in the diagnosis and management of common childhood illnesses is available from Health Child Uganda and has been submitted for publication (Brenner J, personal communication). Based on qualitative data generated through focus group discussions with CHWs, specific operational benefits enabled by mobile phones within the iCCM program have also been described [16]. Herein we detail important steps in system development, training, and implementation of the mobile phone-enabled system as a demonstration of the feasibility of major improvements within the health care system, achievable through coordinated local efforts engaging patients and families, CHWs, district health workers, and academic partners with expertise in child health. This paper may be considered as a guidance document for other researchers and policy makers in Uganda and elsewhere, looking to integrate technology into the existing health care infrastructure.

## Methods

The study of mobile phone support for iCCM in Bushenyi District was funded as a Synergy project extending the work of Healthy Child Uganda [12-15] (a Ugandan Canadian partnership) through the Global Health Research Initiative of the Canadian government (Canadian Institutes for Health Research, International Development Research Centre, and Foreign Affairs, Trade, and Development Canada). Funding was approved in September 2010 and final protocols received ethical approval from Mbarara University of Science and Technology (MUST) in March 2011 and from the Uganda National Council for Science and Technology in June 2011. Ethics approval was also obtained from the University of British Columbia Children’s & Women’s Research Ethics Board and the University of Calgary Conjoint Heath Research Ethics Board in spring of 2011.

The project commenced with gathering and consolidating of all user requirements concerning the CHW program, iCCM Sick Child Job Aids, iCCM Implementation Guidelines, and other relevant literature. The project coordinator (DKT) met with principal investigators and members of the research team to ensure that training and implementation procedures were in line with the approved protocol and that the treatment algorithms were consistent with treatment guidelines. The project technical team was established in July 2011. Details of the iterative process involving technical support, as well as engagement of community health workers and academics, used to develop the treatment mobile phone application were captured. Steps required for the successful implementation of the mobile phone-based system within the iCCM program were defined.

Using a purposefully designed training manual, learning outcomes among CHWs were also captured by research personnel. Specifically, over the five-day training course and during the month post training, CHW performance was monitored. During the implementation phase the number of case report forms submitted by phone was recorded. Finally, as a check on later study procedures, the number of mobile phone-enabled referrals and prevented stock outs was also determined as a quantitative measure of the impact of mobile phones on health service delivery within iCCM. Details of the development and implementation process and training outcomes are presented below.

## Results

The implementation process, including development of algorithms, completion of an environmental scan and resolution of all remaining operational issues in parallel with eventual training of 96 CHWs, took place between July 2011 and March 2012. The seven overlapping phases of the development program are summarized in Table 1.

**Table 1:** Phases* of iCCM + mobile phone project development

Phase 1 Months 1-3	Development of the interface was presented in pilot form at a stakeholders meeting. The interface was tested at the meeting and changes required were recorded.
Phase 2 Month 2	Changes were made to the interface after which the interface was mounted on a regular phone. At a subsequent meeting the application was tested for usability.

Phase 3 Months 1-3	Environmental scan. An environmental scan was completed to look at: 1) common networks used in study area, 2) types of phones commonly used in the study area, 3) mapping of health facilities to determine distance from CHW homes to health centers. Initially it was thought that it might be possible to locate charging facilities at a health centre, which would function as a central recharging point. The environmental scan, however, revealed that health facilities are too far from CHW homes for this plan to be feasible.

Phase 4 Months 2-5	Results from the environmental scan were analyzed and reported. The environmental scan revealed that MTN (a South Africa-based multinational telecommunications company) was the most widely distributed network provider in the study area, and the most common phone was a Nokia. It was determined that each CHW would require his/her own charging system.

Phase 5 Months 4-6	Procurement process: MTN was contacted and negotiations began for procurement of toll free lines and data bundles. Nokia Uganda was also consulted and a price negotiated to purchase 96 Nokia c1-01 phones at 150 000 USH each. The Barefoot Power Company was also identified as a supplier of solar charging systems, from which 96 solar charging systems were purchased. A dedicated server was purchased during this time. Networking and server configuration, domain mounting, and virtualization were completed.

Phase 6 Month 6	The developed and tested application was mapped onto the phones and necessary configurations were made.

Phase 7	Development of an iCCM + mobile phone training manual: Principles considered during development of the manual included 1) integrating the existing iCCM manual with the mobile phone application, 2) usability of the mobile phone, and 3) basic knowledge of mobile phone use.

* Phases overlap to some degree and phase 7 (training manual development) was a continuing process over 9 months

**Figure 1:**
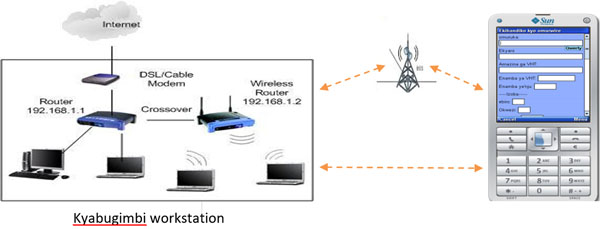
Diagrammatic representation of the interaction between nurses and doctors through the local area network (LAN).

Once all systems and procedures had been validated and streamlined as described in Table 1, a server was established at Kyabugimbi level IV Health Centre in Bushenyi district. The access point was established through the domain name http://www.healthychilduganda.org and this provided the interface for interaction between nurses and doctors through the local area network (LAN) (Figure 1).

**Figure 2:**
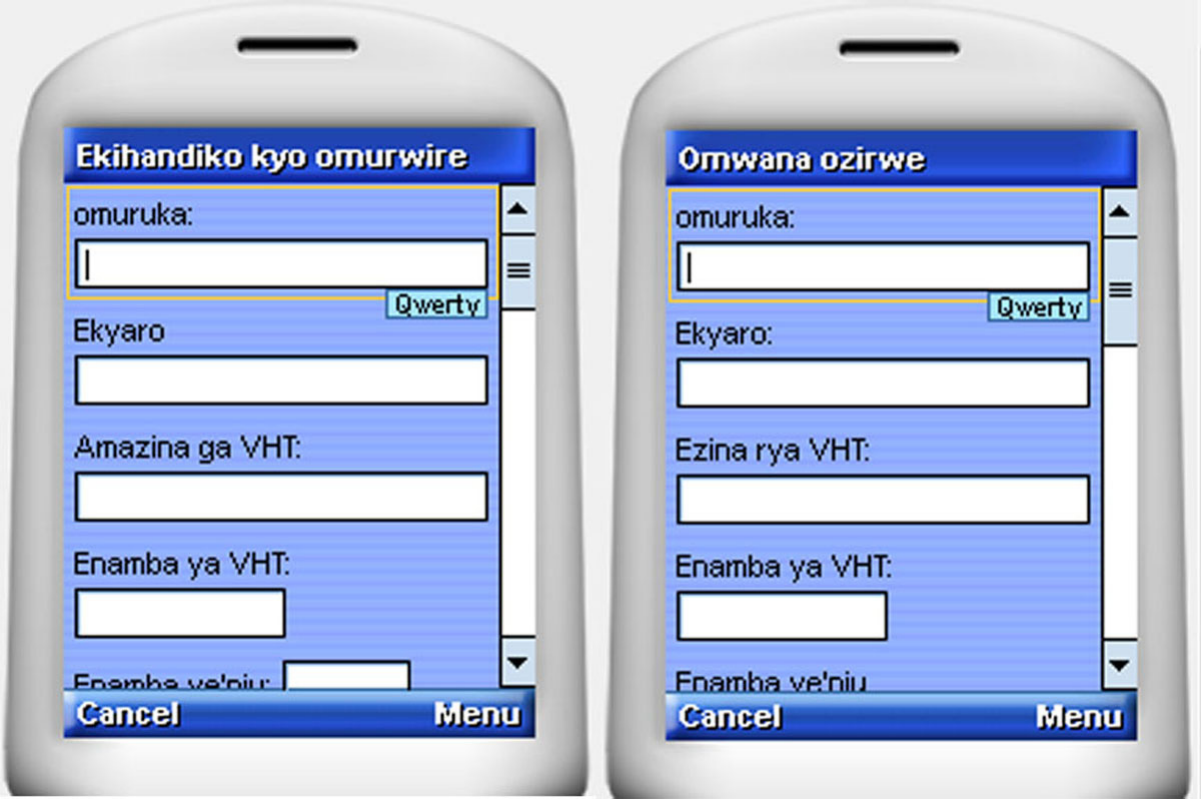
**A** Mobile phone-enabled form for registering newly diagnosed sick children **B** Mobile phone-enabled form for registering newborn babies

The server at Kyabugimbi was virtualized with backup storage at MUST running a Solaris operating system. This technology allows users to consolidate physical data storage. Initially, the system was piloted with 22 CHWs from Ruhumuro Parish interacting with eight health workers. The documentation obtained from this early experience permitted refinement of procedures and development of a user manual^1^. A sample of the screens presented to CHWs is shown here as an indication of ease of use and application of local language (Figure 2).

The Okushaba emibazi screen (Figure 2a) was designed to address concerns about supply chain management for essential drugs used in management of acute childhood illnesses and the CHW was required to enter the following details:

1. Date when requesting drugs

2. Amount of oral rehydration salts (ORS) being requested and amount of ORS remaining

3. Amount of zinc being requested and amount of zinc remaining

4. Amount of amoxicillin red being requested and amount of amoxicillin red remaining

5. Amount of amoxicillin green being requested and amount of amoxicillin green remaining

Data collected under the newborn registration (Omwana Ozirwe) screen (Figure 2b) included the following:

1. Household number

2. Date of registration

3. Name of newborn

4. Name of mother and father

5. Gender of newborn

6. Age of child in days

7. Respiratory rate of child

8. Date of next visit

9. Any signs of disease the child is showing

10. Information on whether the child has been referred to a hospital for treatment.

**Figure 3:**
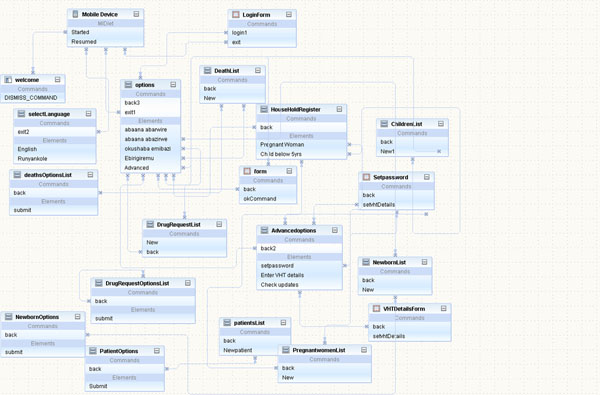
System flow chart illustrating connectivity

The system flow chart presented as Figure 3 illustrates the connectivity that was achieved so that the CHW could access a series of textual manual applications and use short codes or text strings to trigger the server through the telecom company (MTN) base station services network. This design permitted real time connection to a phone-based network using a micro SD card where the application is hosted remotely on the phone with configuration through a global system for mobile communication (GSM) network.

## Discussion

Over a period of nine months, 96 CHWs were trained to a level of competence adequate to support the subsequent community demonstration study that was the main objective of this program [16]. CHWs were trained over an intensive five-day course to be able to make timely diagnoses, recognize clinical danger signs, communicate with higher level healthcare workers about referrals, and initiate treatment in their community with appropriate essential drugs.

Performance by CHWs and the accuracy and completeness of their submitted data was closely monitored in a one-month post training test period before clearance for participation in a subsequent nine-month community trial.

While the development of teaching materials and the training of CHWs generally went smoothly, the process was not free of challenges. Some CHWs were using mobile phones for the first time and required very basic instruction on technique. Some CHWs lacked literacy skills required to fully understand written instructions in English or in local language.

Furthermore, in some communities, at the time of project initiation the introduction of mobile phones, solar chargers, and lighting powered by the charging system led to security issues (i.e. theft) requiring intervention by the local police. With growing appreciation of the value added by the mobile phone-enabled system, security improved.

All other challenges encountered were technical and included the need to revise the entire system flow structure after the initial environmental scan and to write new software. Throughout the development period it proved important to work assiduously with private partners to maintain network connectivity.

Over the course of the eventual study, implementation of the mobile phone service as a complement to iCCM was assessed by quality monitoring of real-time data transmission in case report forms. The number of referrals and correctly treated children, based on the agreed treatment algorithms, was recorded. Births, deaths, and medication stocks in participating communities were also tracked [16].

Trained CHWs proved capable of participating in a community-based research study comparing outcomes in districts served by CHWs trained in iCCM alone and with those who had only routine training in health promotion and disease prevention [16]. CHWs proved capable of mastering all of the required mobile phone skills and no differences were identified relative to previous educational level achieved [16]. The evaluation of CHW performance was conducted primarily at the end of an intensive five-day training course. The ability of CHWs to link with facility health workers (nurses and clinical officers at Kyabugimbi Level IV health centre) was evaluated. The CHWs were cleared for study participation on the basis of their data quality concerning diagnosis, treatment/referral choice, and drug prescription. Direct contact was made by facility health workers with CHWs when remedial intervention was required. Once appropriate knowledge and skills were confirmed, CHWs were considered to be adequately prepared.

The implementation phase of the study served to demonstrate the feasibility of instituting a mobile phone-based support system in Bushenyi District and of employing such a system to improve integrated provision of health care services for children under five at risk. The CHWs proved highly able to master the required technology in order to improve the provision of services to children in their village and expedite referral to appropriate levels of care. Furthermore, based on initial case reports submitted, CHWs demonstrated the capacity to contribute significantly to the timely gathering of data on incidence, referral, and treatment of childhood illness. The quality of the information gathered through the mobile phone network surpassed that obtained from conventional paper records and data retrieval, and analysis from mobile phone records was greatly facilitated.

Perhaps most importantly, the successful implementation of the system confirmed the ability of local information and communication technical personnel to work with community partners, counterparts in the district office of the Ministry of Health, regional health caregivers, and academic partners, to develop user friendly tools for support of CHW decision-making and to make the resulting tools available on a mobile phone platform to the benefit of children in their communities.

The project has also shown the potential for capacity building and empowerment among community health workers without limitation by previous levels of education or preparation [16].

The deployment of mobile phones among CHWs in Bushenyi District exerted important positive effects on the delivery of iCCM and IMCI [16]. In particular, it has been demonstrated that CHWs can be efficiently trained to be able to contribute significantly to the timely gathering of data on incidence, referral, and treatment of childhood illness.

The experience described indicates but does not prove the potential for long term benefit from mobile phone deployment in rural Uganda and similar settings. CHWs, throughout the training program, showed themselves to be willing learners and the skills developed may be anticipated to contribute to operational efficiency and improved accuracy and timeliness of reporting on child health [16]. A functional partnership has been created that is inclusive of village level volunteers, public system health administrators, academic specialists in child health, experts in information and communications technology, and private sector providers. The approach described is sustainable at modest cost but continued application will depend on prioritization decisions to be made by the Ugandan government

## Conclusions

It has proven feasible to develop a prototype mobile phone system for child health applications through the efforts of local information and communications technical personnel, working with health care providers and with academic colleagues, amplifying the iCCM approach advocated by the Ugandan Ministry of Health.

The project described in this paper has fostered an integrated innovation approach that has facilitated public-private partnerships with wireless service providers and a manufacturer of solar chargers. Integration has also occurred among CHWs, health care workers at a district level, university decision-makers, and experts in knowledge translation working in a cooperative fashion with the Uganda Ministry of Health. The operational benefits attained are considered sufficient to add major value to health system efficiency in a rural setting.

Based on the success of this project, training and implementation of a mobile health program could be scaled up with wider targets for intervention in the future. Specifically, a mobile phone system could be expanded to contribute to improved data collection in other health fields and may be particularly useful to enhance antenatal, perinatal, and newborn care [17,18]. By building on the experience described here, further synergies between CHWs and more highly skilled health care workers at level IV health centres and regional hospitals can be promoted.

## List of abbreviations

ARI: Acute respiratory infection; CHW: Community health workers; DFATD: Foreign Affairs, Trade and Development Canada; GHRI: Global Health Research Initiative; GSM: Global system for mobile communication; iCCM: Integrated community case management; IDRC: International Development Research Centre; IMCI: Integrated management of childhood illness; MTN: Mobile Telephone Networks telecom company; MUST: Mbarara University of Science and Technology; ORS: Oral rehydration salts

## Competing interests

The authors declare that they have no competing interests.

## Authors’ contributions

All authors contributed to the planning and execution of the mobile phone project described. DKT prepared the first draft of this manuscript and editing and revision was supervised by JK and SM. All authors have read and are in agreement with the final version.

## Endnotes

^1^ The iCCM/mobile phone training manual described here has been made available on the Healthy Child Uganda website (http://www.healthychilduganda.org). Related study reports [16] will also be posted once published.

## Declarations

The publication costs associated with this article are funded by Foreign Affairs, Trade and Development Canada and the International Development Research Centre through the Global Health Research Initiative.

This article has been published as part of *BMC Health Services Research* Volume 14 Supplement 1, 2014: Uptake and impact of research for evidence-based practice: lessons from the Africa Health Systems Initiative's research component. The full contents of the supplement are available online at http://www.biomedcentral.com/bmchealthservres/supplements/14/S1
